# Anti-Angiogenic Features of Endostatin in Obesity, Liver Fibrosis, and Hepatocellular Carcinoma

**DOI:** 10.3390/biomedicines14030734

**Published:** 2026-03-23

**Authors:** Thomas Grewal, Christa Buechler

**Affiliations:** 1 School of Pharmacy, Faculty of Medicine and Health, University of Sydney, Sydney, NSW 2006, Australia; thomas.grewal@sydney.edu.au; 2 Department of Internal Medicine I, University Hospital Regensburg, 93053 Regensburg, Germany

**Keywords:** obesity, liver fibrosis, angiogenesis, hepatocellular carcinoma

## Abstract

**Background/objectives**: Endostatin is a cleavage product of collagen XVIII and a potent anti-angiogenic factor. Angiogenesis is essential for adipose tissue growth and contributes to liver fibrosis and cancer, suggesting a potential therapeutic role for endostatin in obesity, chronic liver diseases, and hepatocellular carcinoma (HCC). This review article summarises published data on the role and expression of endostatin in obesity, liver injury, and HCC. **Methods**: PubMed and Google databases were searched using the terms “endostatin and liver”, “endostatin and HCC”, “endostatin and obesity”, and “endostatin and adipose”. Studies published in peer-reviewed journals relevant to this review were considered and reviewed for valuable insights. **Results**: Endostatin is much more than an inhibitor of angiogenesis; it exerts direct effects on adipocytes and myofibroblasts. Endostatin inhibits adipose tissue growth, and studies using Endostar—a modified form of endostatin approved in China for treating lung cancer—have demonstrated its protective effect in liver fibrosis. However, other studies have shown that endostatin activates hepatic stellate cells, indicating a role in tissue regeneration. Most research on endostatin has focused on cancer, and animal and human studies have shown the benefits of Endostar therapy in HCC. **Conclusions**: Endostar is a promising treatment for HCC and may also become an attractive drug for liver fibrosis. Hence, angiostatic therapy is not without risks and may only be suitable for selected patients.

## 1. Introduction

Adipose tissue functions as a highly active endocrine organ and plays a critical role in whole body lipid metabolism, consistently interacting with the liver, the central organ in lipid homeostasis [1,2]. Adipocytes store significant quantities of triglycerides and cholesterol, which underscores their pivotal role in overall lipid metabolism [3]. In the obese, dysfunctional adipose tissue cannot appropriately store excess amounts of triglycerides, which are then deposited in other tissues such as the liver and muscle, thereby impairing the proper function of these organs [4,5].

In adults, the formation of new adipocytes is a normal aspect of adipose tissue turnover. In obesity, hyperplasia (the appearance of new adipocytes) and cell hypertrophy contribute to adipose tissue expansion [6]. The growth of adipose tissue through hyperplasia is regarded as a more advantageous mechanism than adipocyte hypertrophy, which is linked to hypoxia, immune cell infiltration, and potentially adipose tissue fibrosis [7,8,9].

Excessive growth of adipose tissue is associated with obesity and is generally considered harmful to human health [10,11,12]. However, excess fatty acids are safely stored in growing subcutaneous adipose tissue, thereby protecting against lipotoxicity [5]. This illustrates that not the accumulation of functional adipose tissue, but malfunctioning fat tissue resulting from excessive hypertrophic growth critically contributes to disease progression. This is also evident in the abnormal adipokine release from adipose tissue in obesity [13,14,15].

Leptin is a well-studied adipokine that was initially discovered as a satiety hormone. In obese individuals, blood leptin levels are elevated, and leptin resistance contributes to hyperleptinemia and impaired appetite control [16]. Leptin-deficient ob/ob mice are extremely obese and develop type 2 diabetes. Leptin stimulates lipolysis and fatty acid oxidation in adipocytes and, moreover, increases adipogenesis. The injection of leptin led to a reduction in both adiposity and the amounts of white and brown fat [17]. Leptin has fibrotic effects, and neutralizing leptin antibodies protects from fibrosis in the heart, blood vessels, kidney, lung, and liver [18].

Conversely, serum adiponectin levels decrease in obese individuals. Adiponectin is primarily produced by adipocytes and exerts numerous beneficial effects in peripheral tissues [2,19]. Furthermore, adiponectin acts as an autocrine factor in adipose tissue, promoting preadipocyte proliferation and adipogenesis [20]. In so-called “metabolically healthy obesity”, it is suggested that the remodelling of the extracellular matrix (ECM) occurs in a manner required for the healthy expansion of subcutaneous fat depots [21,22]. Metabolically healthy obese individuals had higher adiponectin levels than metabolically unhealthy obese subjects, further indicating a role for this adipokine in the healthy growth of adipose tissue [20,23].

Adipose tissue can be broadly categorised as white and brown fat. Brown fat contains many mitochondria and dissipates energy to produce heat. This type of fat is considered healthy, and various approaches are being explored to stimulate its activity/growth to induce weight loss [24,25]. White adipose tissue is mostly found in subcutaneous and visceral fat depots. The latter is located within the peritoneal cavity and surrounds internal organs, such as the intestine or the liver [20]. Excess visceral fat is considered harmful and is associated with metabolic diseases such as type 2 diabetes, metabolic syndrome, and metabolic dysfunction-associated steatotic liver disease (MASLD) [20,26]. Visceral fat differs from subcutaneous fat with respect to the adipokines it releases. For instance, the high production of interleukin (IL)-6 by visceral adipose tissue has been shown to contribute to metabolic dysregulation in obese individuals [27]. Obesity is a scourge of our time, in particular in Western nations, and attempts to reduce body fat are urgently needed. One therapeutic approach aims to promote the conversion of white to beige adipocytes. This process is called ‘browning’, in which beige adipocytes burn energy to produce heat, similar to brown fat [28].

As outlined above, enlargement of adipose tissue can occur through hypertrophy of existing adipocytes or hyperplasia [20]. Tissue growth requires adequate oxygen and nutrient supply, and angiogenesis is essential to ensure the transportation of vital substances by blood vessels, including cytokines, hormones, and growth factors [29]. Angiogenesis is a highly coordinated process involving endothelial cell proliferation, migration, invasion, and formation of new blood vessels [29]. Inadequate angiogenesis combined with hypertrophic adipocyte growth induces cell death, a trigger for adipose tissue inflammation and fibrosis. Consequently, adequate lipid storage in this tissue is compromised, further contributing to fatty acid deposition in peripheral tissues and insulin resistance [20].

Adipocytes secrete pro-angiogenic molecules such as vascular endothelial growth factor (VEGF), and their overexpression in adipocytes increases vascularization. Moreover, VEGF-A induces browning of white adipose tissue, which is associated with upregulation of uncoupling protein 1 (UCP1) and increased energy expenditure. This protects against obesity-induced metabolic dysregulation [30]. However, elevated levels of VEGF-C, a growth factor relatively specific to the lymphatic vascular system, have been associated with weight gain and insulin resistance [31].

Adipocytes not only secrete pro-angiogenic molecules such as VEGF, tumor necrosis factor (TNF), and IL-6, but also produce anti-angiogenic factors [29,32]. Endostatin is an anti-angiogenic protein derived from collagen XVIII upon cleavage by cathepsin L, S, or matrix metalloproteinases (MMPs). This 20 kDa protein is homologous to the C-terminal end of collagen XVIII [33]. Collagen types, including collagen XVIII, are widely expressed and function as structural components of basement membranes. Collagen XVIII supports preadipocyte differentiation and the maintenance of the differentiated state. A deficiency in collagen XVIII results in reduced adiposity, liver steatosis, and hypertriglyceridemia in mice [21,34]. Mutations in this gene cause Knobloch syndrome, an autosomal recessive condition characterized by degeneration of the vitreoretinal and macular regions of the eye, as well as an occipital encephalocele [35].

Endostatin has been found primarily in hepatocytes but has also been identified in 3T3-L1 adipocytes [32] and binds to a variety of proteins and cell-surface receptors. In particular, endostatin has been shown to interact with VEGF receptors 1 and 2, thereby inhibiting VEGF-A binding to these receptors, which are required for angiogenesis and vascular permeability [33,36]. Furthermore, endostatin binds to the α5β1 fibronectin receptor, thereby preventing binding of the angiogenic ligand fibronectin [33,37]. In addition, endostatin exhibits anti-angiogenic activity through multiple mechanisms, including inducing apoptosis in endothelial cells, inhibiting endothelial cell proliferation and migration, and suppressing angiogenic inducers and signaling pathways [33,38].

The angiogenesis-inhibiting effect of endostatin is exerted by monomeric endostatin, whereas its trimeric form is a motility-inducing molecule in endothelial cells [39]. Proteolysis in the hinge region of collagen XVIII converts endostatin from a trimerized configuration into a monomeric form [40]. Angiogenesis is essential for tissue growth, and the multiple anti-angiogenic effects have made endostatin a promising drug target in cancer therapy [33]. Angiostatic molecules, such as endostatin, have been studied for their potential to prevent obesity and associated metabolic complications [41]. Endostatin also exerts anti-inflammatory and anti-fibrotic effects, suggesting potential as a treatment for various other diseases, including chronic liver diseases [33]. This review article summarises studies on the function and expression of endostatin in obesity, with a focus on its role in adipose tissues. Given the close association between obesity and chronic liver diseases, a chapter focusing on the role of endostatin in chronic liver disease and hepatocellular carcinoma (HCC) has been added. The latter commonly arises from metabolic dysfunction-associated steatohepatitis (MASH) in industrialized countries [4,42,43,44,45,46]. Hence, studies that measured endostatin levels in the blood to assess its potential as a biomarker for chronic liver diseases and HCC are also described.

## 2. Endostatin and Obesity

**Figure 1:**
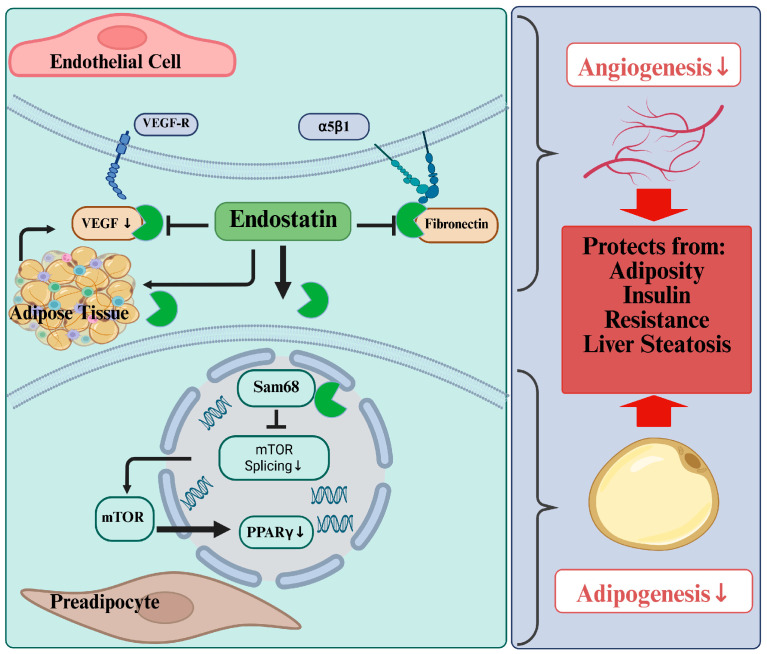
The function of endostatin in adipose tissue. Multiple activities of endostatin inhibit angiogenesis and adipogenesis, and protect against obesity, insulin resistance, and liver steatosis. Endostatin hinders the binding of vascular endothelial growth factor (VEGF) and fibronectin to their respective receptors, thereby inhibiting angiogenesis. It also reduces VEGF secretion from adipose tissue. In preadipocytes, endostatin binds to Sam68, preventing the accurate splicing of mechanistic target of rapamycin (mTOR) mRNA and leading to mTOR dysfunction. This reduces mTOR’s ability to stimulate adipogenesis by activating peroxisome proliferator-activated receptor γ (PPARγ). Created in BioRender. Pollinger, K. (2026) https://BioRender.com/z3x9au6 (accessed on 12 March 2026).

Mutations in the gene responsible for leptin production underlie excessive eating and severe obesity in the ob/ob mouse strain [47]. Strikingly, injecting recombinant endostatin expressed in *Escherichia coli* caused a 5% weight loss in ob/ob mice, whereas the control group gained weight [48]. Endostatin treatment had no effect on appetite, indicating that anti-angiogenic therapies reduce adipose tissue mass rather than food uptake [48] (Figure 1).

In another study, recombinant endostatin expressed in *Escherichia coli* was shown to prevent diet-induced obesity. This study provided evidence that the anti-adipogenic activity of endostatin could be explained by its binding to the Sam68 RNA-binding protein [38], a key regulator of mechanistic target of rapamycin (mTOR), which controls adipocyte differentiation. Sam68 binding to endostatin blocks its interaction with intron 5 of the mTOR pre-mRNA, impairing splicing and subsequently reducing mTOR protein and function. mTOR positively regulates adipogenesis, partly by activating the peroxisome proliferator-activated receptor (PPAR) γ, a central transcription factor for adipocyte differentiation [38,49] (Figure 1). Blockage of mTOR signaling by endostatin protected against obesity, insulin resistance, and liver steatosis [38] (Figure 1). This suggests that the loss of fat mass in endostatin-treated rodents is linked to pathways that promote the elimination of excess lipids, thereby preventing their deposition in peripheral organs, such as the livers, of these animals.

In addition, others reported that endostatin reduced levels of secreted VEGF protein and *VEGF-A* mRNA expression in adipose tissue [32] (Figure 1). These latter observations are linked to studies demonstrating that VEGF-A/VEGFR2 blockade in adipose tissue reduces body weight gain and improves metabolic health [30]. Repression of VEGF induces browning of white adipocytes and increases energy expenditure, thereby reducing adipose tissue mass [50].

A novel multitarget fusion protein, referred to as AARP (CTT peptide-endostatin mimic-kringle 5 protein), comprises an MMP-2/9-selective inhibitory peptide, the N-terminal 25 amino acids of endostatin, and the Kringle 5 fragment of human plasminogen—a further anti-angiogenic agent. This molecule was designed to simultaneously inhibit the enzymatic activity of MMP-2/9 and angiogenesis for cancer therapy. Mice fed a high-fat diet and treated with AARP showed less fat, smaller adipocytes, reduced blood vessel density in adipose tissues, and an improved insulin response [51]. In the drug-treated mice, leptin, resistin, and adiponectin levels normalised, and plasma levels of free fatty acid, triglyceride, and cholesterol all declined [51]. Furthermore, AARP treatment normalized excess hepatic fat storage and the expression of genes such as sterol regulatory element-binding protein (SREBP)-1c and fatty acid synthase (FAS), both of which are important for fatty acid synthesis. Also, aminotransferase levels as a marker of liver injury normalized with AARP treatment. Other benefits of AARP treatment included normalisation of *FAS* mRNA levels in subcutaneous tissue and *MMP-2/9* in epididymal tissue. Interestingly, *UCP1* expression was strongly induced in the brown fat and the white subcutaneous fat [51]. Thus, at least in mice on a high-fat diet, AARP appears to increase energy expenditure, thereby reducing adipose tissue mass and preventing excessive lipid storage in peripheral organs [51].

Anti-angiogenic therapies were found to not only lower adipose tissue mass but also improve metabolic health. Suppressing VEGF promoted enlargement of brown adipose tissue and stimulated the formation of brown-like adipocytes within white adipose tissue, accompanied by increased energy expenditure [50,52].

The kringle 5 fragment of human plasminogen inhibits endothelial cell proliferation and angiogenesis [53], but its influence on adipose tissue development has not been studied. Adipose tissue growth requires MMPs to remodel the extracellular matrix, and is associated with higher collagen levels and blood vessel density in adipose tissues [54]. Blockage of MMP2 and MMP9 alone had only marginal effects on adipose tissue growth and density of blood vessels in high-fat diet-fed mice [55]. These previous results suggest that the MMP blocking domain of AARP plays a minor role in its beneficial metabolic effects [51]. Primarily reduced VEGF levels and inhibition of angiogenesis seem to account for most of AARP’s effects on metabolic health [51].

It should be noted here that other well-studied adipokines also regulate angiogenesis [56]. Adiponectin, a hormone produced almost exclusively by adipocytes, exhibits anti-angiogenic properties, including the inhibition of endothelial cell proliferation and migration [56]. There is also evidence that adiponectin can promote angiogenesis following ischaemic injury. The mechanisms underlying the opposing effects of adiponectin remain unclear, but its different isoforms, found in low-, middle-, and high-molecular-weight complexes, appear to exert opposing effects [56]. In adipose tissue, adiponectin promotes hyperplastic growth by inducing adipogenesis and stimulating angiogenesis, thereby preventing hypoxia in growing fat deposits [57]. Leptin, elevated in the serum of obese individuals, also stimulates angiogenic processes [56]. Leptin activates extracellular signal-regulated kinase (ERK) 1/2, enhances endothelial cell viability, and upregulates the expression of pro-angiogenic molecules, such as MMPs, VEGF, and its receptor VEGFR1 [56].

Endostatin was found to affect gene expression in the epididymal adipose tissue of female mice two hours after injection, an effect that occurred only when endostatin was injected during the day (vs. night). In these studies, *PPARγ*, *SREBP1*, *CCAAT/enhancer-binding protein alpha*, *fatty acid-binding protein 4*, *perilipin 2*, *hormone-sensitive lipase*, *monoglyceride lipase*, and *FAS* expression levels were reduced following endostatin injection. This analysis suggests that endostatin reduces adipogenesis, fatty acid synthesis, and lipolysis in fat depots [58].

When Endostar (a recombinant endostatin) was injected once daily for 10 days into tumor-bearing mice, they gained less body weight than the control group [59]. In contrast, treatment of glioblastoma-bearing mice with Endostar for 15 days had no effect on body weight [60]. Currently, the in vivo effect of endostatin on adipose tissue mass has not been studied in great detail, and Endostar-related studies were predominantly performed in tumor-bearing mice or patients with different cancers [59,61,62,63], making it difficult to monitor and draw conclusions on its effect on body weight, which is affected by cancer [64].

While it is interesting that blocking angiogenesis reduces adiposity, the approaches listed above are unlikely to be suitable for common weight-loss therapies, given angiogenesis’s essential role in the body [29,49]. The possibility of using adipose tissue-specific applications as an alternative approach has not been studied.

## 3. Endostatin Levels in the Blood and Association with Sex, Age, and Body Mass Index

Endostatin is present at high concentrations in blood, with mean levels of around 120 ng/mL reported in an early study [65]. Levels of endostatin in the blood and liver of mice exhibited a subtle daily rhythm [58], peaking during the day [58], suggesting that the timing of blood collection modestly affected serum endostatin concentrations. However, it is not known whether this also applies to humans.

Sex can be a confounding factor in systemic protein levels and tissue protein expression, but most studies do not account for sex-specific differences [66]. While several studies did not observe sex-specific differences in circulating endostatin levels [65,67], one study reported higher serum endostatin levels in women, both in the control group and in patients with type 2 diabetes [68] (Table 1). Studies comparing tissue endostatin expression between male and female rodents or patients are lacking, to our knowledge.

Age could also be important when analysing serum endostatin levels, as circulating endostatin levels were higher in older mice. Endostatin expression in the kidney was approximately 6-fold higher in aged animals [71]. However, no association was observed between serum endostatin levels and age in healthy young adults [67] (Table 1). Serum levels in patients older than 65 years were much higher than in those aged < 65 years [69] (Table 1). Alike the impact of gender, the effect of age on endostatin levels in blood and tissues remains understudied.

Given that endostatin protects against obesity [38], it was hypothesised that overweight/obese individuals would display low systemic endostatin levels. However, a positive correlation was observed between blood endostatin levels and body mass index in both sexes. Furthermore, a significant increase in endostatin levels was observed in overweight and obese females compared to normal-weight women [65] (Table 1). Among 5026 randomly chosen middle-aged participants enrolled in the Swedish CardioPulmonary BioImage Study, plasma endostatin was positively associated with the waist circumference [70] (Table 1).

Contrary to findings that blood endostatin levels change with obesity, plasma endostatin levels remained unchanged in patients before bariatric surgery and at 12 or 48 months after, during which body mass index decreased significantly. Mean levels were approximately 160 ng/mL prior to surgery and 190 ng/mL at the end of the study [72]. Insulin resistance and systemic C-reactive protein levels significantly improved over the 48-month follow-up period [72].

However, lower serum endostatin levels in both sexes have been reported in patients with type 2 diabetes. The participants with diabetes in this study had a higher body mass index than controls, although the difference was not statistically significant [68]. Heart rate and blood pressure of the diabetic patients were normal, while kidney function had not been evaluated [68]. Hence, endostatin levels may decline in patients with type 2 diabetes independent of obesity.

In this context, it should be noted that angiogenic molecules, such as VEGF and angiogenin, are elevated in obese individuals [56], and the angiogenic-to-angiostatic protein ratio may provide greater insight into angiogenesis in obesity. However, a meta-analysis found that VEGF-A was not elevated in obese individuals but was associated with hyperglycemia, highlighting the complex association between obesity, metabolic diseases, and angiogenesis [73]. In mice, endostatin did not significantly alter blood glucose levels [58], and further study is needed to evaluate whether there is any association of endostatin and blood glucose levels in humans. In the Swedish CardioPulmonary BioImage Study, plasma endostatin was not associated with diabetes [70].

Renal impairment and hypertension are associated with elevated blood endostatin levels [70,74] (Table 1) and may confound observational studies. To sum up, there is no evidence of a strong association between blood endostatin levels and sex or body weight, compared with classical adipokines such as leptin and adiponectin, which are consistently higher in female blood and are increased/decreased in obesity [75,76].

Given that exercise commonly improves metabolic health, its effects on VEGF and endostatin were tested in rats with coronary artery disease [77]. Following isoproterenol administration, the rats underwent continuous and non-continuous aerobic exercise on a treadmill for eight weeks. While VEGF levels in the blood increased in both groups, endostatin levels did not change significantly, although it remained unclear whether exercise reduced body weight in this study [77]. Physical stress from ergometry increased serum endostatin levels in both sexes of type 2 diabetics and controls [68]. Whether this is a beneficial effect of exercise, lowering body fat mass in the long term, requires further research.

There is surprisingly very limited insight into the tissue expression patterns of endostatin in obesity. In rats fed a high-fat diet, endostatin protein levels increased in retroperitoneal white adipose tissue and decreased in the liver compared to animals fed a standard diet [78]. Endostatin levels in the brown fat of lean and overweight rats were similar [78]. The main sources of collagen XVIII in the rat liver are hepatocytes and bile duct epithelia [79]. Because primary mouse hepatocytes secrete endostatin [58], the liver appears to be a major source of endostatin in the circulation. Reduced hepatic endostatin levels in obese rats may therefore suggest that blood endostatin levels decline in parallel with hepatic levels [78]. Indeed, higher hepatic and serum endostatin levels were reported in patients with liver fibrosis [80], underscoring that endostatin in blood is mostly derived from the liver.

## 4. Endostatin and Liver Fibrosis

Obesity is a risk factor for the progression of chronic liver diseases, such as hepatitis C and B virus infection and alcoholic liver disease, as well as for the development of MASH and HCC [81]. In fact, deregulated cytokine and adipokine release from adipose tissue in obesity has been shown to contribute to the progression of chronic liver diseases [2,81,82]. Leptin promotes hepatic inflammation and enhances collagen deposition in the liver [83], whereas adiponectin, which declines in obesity, protects against liver fibrosis [2,84]. Endostatin prevents fat accumulation and normalises adipokine levels in obese rodents [51], suggesting that endostatin protects against MASLD. To our knowledge, this hypothesis has not yet been tested. Furthermore, endostatin is an anti-angiogenic protein [33], and angiogenesis is closely linked to the progression of chronic liver diseases, such as MASLD. It has been suggested that therapies involving anti-angiogenic factors could impede the progression of fibrosis [85].

Transforming growth factor (TGF)-β is the central cytokine for the initiation and progression of liver fibrosis. In hepatic stellate cells (HSCs), TGF-β induces the production of ECM proteins and inhibits MMP expression, while enhancing the expression of tissue inhibitors of metalloproteinases (TIMPs) to prevent ECM degradation. This cytokine activates quiescent HSCs, which then become myofibroblasts characterised by α-smooth muscle actin (α-SMA) and ECM protein expression [86,87,88].

**Figure 2:**
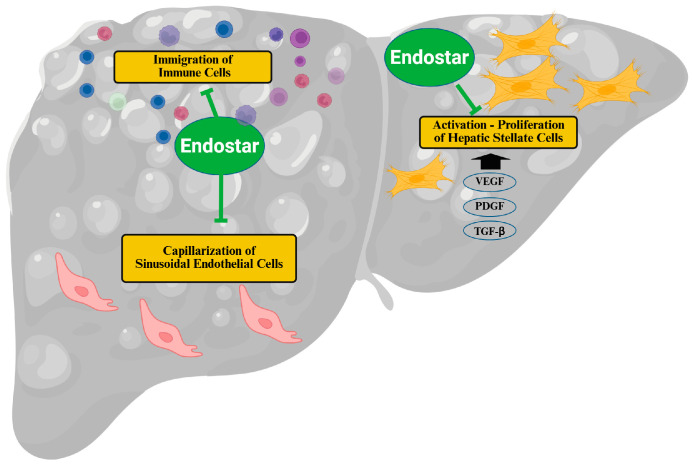
The role of Endostar, a recombinant endostatin, in liver fibrosis. Endostar hinders the activation and proliferation of hepatic stellate cells, induced by vascular endothelial growth factor (VEGF), platelet-derived growth factor (PDGF), and transforming growth factor (TGF)-β. On the other hand, Endostar reduces the infiltration of inflammatory cells into the injured liver. Furthermore, Endostar prevents the capillarization of sinusoidal endothelial cells. These effects reduce and even reverse liver fibrosis. Created in BioRender. Pollinger, K. (2026) https://BioRender.com/x823yw7 (accessed on 12 March 2026).

In the event of liver injury, hepatocytes produce VEGF, which stimulates the activation and proliferation of HSCs via VEGF-receptors 1 and 2. This results in enhanced production of ECM proteins and TGF-β. As endostatin inhibits VEGF signaling and angiogenesis, it was assumed to have a protective role in HSC activation [89]. Indeed, co-treatment with the recombinant endostatin Endostar normalised VEGF-induced levels of collagen 1, TGF-β, and α-SMA in the HSC-T6 cell line [90] (Figure 2 and Table 2). When these cells were incubated with TGF-β1 or platelet-derived growth factor-BB (PDGF-BB), pretreatment with Endostar for 1 h inhibited cell proliferation and ECM protein expression and blocked Ras homolog gene family, member A/rho-associated protein kinase 1 signaling pathways, which were induced by profibrotic agonists [91] (Figure 2 and Table 2). The HSC-T6 cells from Sprague-Dawley rats express α-SMA, a marker of activated cells [92], indicating that endostatin reversed this profibrotic stage.

In primary HSCs, endostatin activated the expression of α-SMA and collagen 1A1 (Table 2). In these cells, recombinant endostatin activated focal adhesion kinase and ERKs downstream of integrin α5/β1 [80]. Cultivation of primary HSCs on plastic dishes for several days activates the cells and induces expression of ECM proteins [93]. The primary HSCs used in this study were cultured for only 8 h, which may not have been sufficient to activate the cells [80,94]. In these cells, endostatin strongly induced the expression of profibrotic genes [80] (Table 2). Thus, contradictory and inconsistent results regarding HSC activation by endostatin have been reported, which may be explained by different experimental settings [80,90,91]. From these studies, it appears that endostatin antagonizes the effects of TGF-β and PDGF-BB on the expression of fibrotic genes and proteins in activated HSCs. In quiescent cells, endostatin may even increase the expression of these genes, thereby contributing to myofibroblast activation and fibrosis [80,90,91].

It should be noted that the studies showing antifibrotic effects in HSCs both used Endostar, which was purchased from the same company [90,91]. However, in one study, 5 µg/mL, whereas up to 500 µg/mL Endostar was used in the second one [90,91] (Table 2). This recombinant protein consists of 183 amino acids of the C-terminal part of collagen XVIII, a nine amino acid long N-terminal peptide encoding a histidine-tag and an additional N-terminal tag to increase protein stability. Moreover, pre- and co-incubation of the profibrotic agents with endostatin, as well as treatment of cells with endostatin alone have been examined. HSCs most likely do not produce endostatin [79], thereby excluding this as a confounding factor. The study showing activation of HSCs by endostatin used relatively low concentrations [80,90,91], and endostatin’s concentration-dependent effects have been described [95].

Unlike monomeric endostatin, which exerts potent anti-angiogenic effects, the trimeric form derived from the C-terminal domain of collagen XVIII promotes a pro-migratory phenotype in endothelial cells. Notably, this pro-migratory activity is counteracted by the presence of monomeric endostatin [96]. Endostar is a monomeric protein, and this most likely also applies to endostatin used by Zuo et al. [80].

The anti-fibrotic effect of Endostar was also demonstrated in primary human skin fibroblasts. Here, the cells were treated with 200 ng/mL PDGF-BB or 10 ng/mL TGF-β1 for 72 h, and 5 μg/mL Endostar was added at the 24th hour. Endostar dramatically reduced the overexpression of collagen I, hydroxyproline, and α-SMA caused by TGF-β1 and PDGF-BB treatment [97]. Endostar suppressed TGF-β1 and PDGF-BB inducible PDGF-receptor and phosphorylated (active) ERK (p-ERK) levels, while total ERK expression was unaffected [97]. These results suggest that endostatin may exert similar effects on primary cells and cell lines, but only has an anti-fibrotic effect on activated cells. However, recombinant endostatin at doses of 300 and 3000 ng/mL increased the proliferation of α-SMA-positive primary cardiac myofibroblasts in rats, contributing to healing after myocardial infarction [98]. This shows that cell activation and the use of primary versus transformed cells cannot explain the contradictory effects of endostatin.

Studies using Endostar have shown antifibrotic effects in myofibroblasts [90,91]. Endostar is more stable and resistant to proteolysis than unmodified endostatin [99], which may be further processed in cell culture medium to produce profibrotic fragments, a hypothesis to be experimentally verified. The integrity of the N-terminal end of endostatin is crucial for its activity [100], another study showed antifibrotic effects of its C-terminal peptide [101], and N/C-terminal deletion mutants were also found to exhibit anti-angiogenic effects [102]. Which parts of this molecule exert antifibrotic activity in HSCs have not been finally evaluated.

The role of endostatin was also investigated in animal models. A 47 amino acid long peptide derived from the C-terminus of endostatin (E4) prevented fibrosis in the bleomycin-induced pulmonary fibrosis model [101]. Recombinant endostatin also exhibited protective properties in this model, inhibiting VEGF and VEGFR2 expression, as well as ERK1/2 activation. Furthermore, alveolar type II cell apoptosis decreased, the number of infiltrating immune cells declined, and levels of TNF and TGF-β decreased [103]. The E4 peptide was also found to inhibit TGF-β- and bleomycin-induced dermal fibrosis [104]. Notably, the E4 peptide reversed established fibrosis, which could have significant clinical value. This peptide decreased levels of early growth response gene-1, a transcription factor that mediates the effects of multiple fibrotic triggers, and lysyl oxidase, an enzyme that cross-links collagen [104].

In mice with CCl4-induced liver fibrosis, Endostar was administered concurrently with CCl4 and was associated with lower hepatic expression of α-SMA, collagen I, TGF-β, and VEGF receptors 1 and 2, as well as lower aminotransferase levels. Hepatic inflammation and fibrosis were improved [90] (Table 2 and Figure 2). Endostar also affected liver sinusoidal endothelial cells, which line the liver sinusoids [105]. In early liver injury, liver sinusoidal endothelial cells experience phenotypic changes, including the development of a continuous basement membrane, a process known as capillarization [105]. Capillarization of liver sinusoidal endothelial cells appears to permit HSC activation; however, it remains uncertain whether this process directly drives or promotes HSC activation [106]. Endostar reduced capillarization of liver sinusoidal endothelial cells and ameliorated hepatic inflammation in a mouse model of CCl4-induced liver fibrosis [107] (Figure 2). Endostar treatment reduced VEGFR1/2 protein levels in the liver, suggesting that inhibition of the VEGF signaling pathway contributes to Endostar’s protective effects [107].

In chronic liver injury, reduced VEGF signaling leads to capillarization of sinusoidal endothelial cells, suggesting that lowering VEGF signaling via Endostar is not the underlying pathway, at least for these cells [108]. However, VEGF exemplifies context-dependent signaling. In healthy tissue, it functions as an essential survival factor, helping maintain vascular integrity [109], but within tumors, it promotes aberrant, highly disorganized angiogenesis [110]. Under physiological conditions, low-level homeostatic paracrine VEGF signaling sustains stable, differentiated vasculature—particularly in liver sinusoidal endothelial cells. In pathological settings, however, elevated concentrations and combined autocrine/paracrine signaling drive uncontrolled vascular growth [109,110]. VEGF is also essential for tissue repair and the resolution of fibrosis, further illustrating its dichotomous role [111].

A further study could not provide evidence for an anti-fibrotic role of endostatin. These experiments did not observe an effect of endostatin on sinusoid capillarization. Injection of recombinant endostatin into mice challenged with CCl4 did not improve liver fibrosis [80]. Endostatin is produced through cathepsin S cleavage of collagen XVIII, and levels of active cathepsin S were induced during liver injury. Macrophages were the main source of cathepsin S in the injured liver [80]. In cathepsin S-deficient mice, which were protected from liver disease in the CCl4 model, recombinant endostatin reversed the protective effects of cathepsin S deficiency, restoring the expression of profibrotic molecules [80].

This study supported the concept that endostatin can promote fibrosis, consistent with its profibrotic effects on primary HSCs [78], yet it remained unclear why this was observed only in mice lacking cathepsin S and not in controls [80]. Similarly, transgenic expression or continuous infusion of recombinant endostatin in mice led to renal fibrosis at a young age [112]. The dual role of endostatin as both a potent profibrotic [112] and an anti-fibrotic molecule [90] shows that angiostatic therapies in patients with liver fibrosis must be carefully considered.

## 5. Circulating and Hepatic Endostatin Levels in Liver Disease

A few studies have analysed endostatin expression in experimental models of liver injury. In mice treated with CCl4, hepatic and serum endostatin levels were increased [80]. It has also been reported that mice treated with CCl4 and those without cirrhosis had similar serum endostatin levels [113], which did not support the use of serum endostatin as a non-invasive marker for cirrhosis. This finding is consistent with unchanged expression of collagen XVIII in fibrotic livers, which, unlike procollagen α1(I) and TIMP-1, did not change during fibrogenesis. Collagen XVIII was slightly upregulated in rats with biliary fibrosis, in contrast to the significantly higher upregulation of procollagen α1(I) and TIMP-1 transcript levels [79].

In patients with HCC, hepatic collagen XVIII/endostatin in non-tumour tissues did not correlate with the extent of fibrosis. This study used immunohistochemistry, which cannot distinguish collagen XVIII from endostatin [114].

Due to the limitations of current non-invasive biomarkers for diagnosing liver fibrosis, new markers are still being sought [115]. However, endostatin in the blood is certainly not a standalone diagnostic tool (Table 3).

One study provided evidence that serum endostatin levels are regulated by the spleen, demonstrating that the spleen contributed to hepatic cathepsin S levels, which cleaved collagen XVIII to increase endostatin production in the fibrotic liver [80]. Conversely, splenectomy in mice was found to reduce serum levels of cathepsin S and endostatin [80]. Paired serum samples from patients with liver cirrhosis, taken before and after splenectomy, showed decreased serum cathepsin S and endostatin levels within one month of surgery in the latter group. However, the FibroScan score did not differ between these time points, indicating that spleen, rather than liver disease severity, plays a significant role in endostatin production [80]. Patients with compensated liver cirrhosis have a smaller spleen than those with decompensated disease [121], but both cohorts had similar serum endostatin levels compared to patients with chronic hepatitis [116] (Table 3). Accordingly, serum endostatin levels were not correlated with the Child-Pugh score in cirrhosis [116]. Furthermore, serum endostatin levels were not increased in patients with HCC compared with controls, and cirrhotic patients with and without HCC had plasma endostatin levels comparable to those of controls [116,117,122] (Table 3). Poon et al. observed a modest decline in endostatin levels of HCC patients with cirrhosis compared to those with non-cirrhotic disease [117]. However, others reported higher serum endostatin levels in patients with HCC than in controls, with no difference between patients with liver cirrhosis and healthy controls [118] (Table 3). Another study reported higher serum endostatin levels in HCC than in liver cirrhosis, hepatitis, and healthy controls, with levels similar across the latter three cohorts [119] (Table 3).

Surprisingly, serum endostatin levels were higher in HCC patients than in controls and increased further after surgery [123]. Infection with the hepatitis C or B virus was associated with higher blood endostatin levels than in healthy controls. These levels were further increased in patients with HCC [120] (Table 3).

The patient cohorts described in this paragraph all included more males than females and comparable patient ages (Table 3) [116,117,118,119,120]. For analysis of serum endostatin, different ELISA assays were used, and levels ranged from 1 ng/mL up to 200 ng/mL. Based on current data, serum endostatin is elevated in HCC but is not consistently altered in liver cirrhosis. However, the patient populations had mixed disease etiologies, and whether serum endostatin is associated with these etiologies has not been evaluated [116,117,118,119,120]. Portal hypertension, splenomegaly, and hepatorenal syndrome are common complications of liver cirrhosis [124,125]. Renal impairment and hypertension are associated with elevated blood endostatin levels [70,74], and splenectomy reduced blood endostatin levels [80]. All of these factors must be considered when examining the association between blood endostatin levels and fibrosis. Furthermore, HCC can develop in a non-cirrhotic liver [126], making comparisons with cirrhotic patients invalid. There are differences in common liver diseases between Asia and Europe/North America [127], but most of the above-described studies were conducted in Asia (Table 3).

A study from Egypt found that patients with chronic HCV and minimal fibrosis had an average endostatin level of 112 ± 30.4 ng/mL, and those patients with significant fibrosis had an average endostatin level of 168 ± 43.8 ng/mL in serum [128]. In this study, a score was developed based on Fas/CD95, hepatocyte growth factor, endostatin, platelet, and albumin levels to non-invasively diagnose fibrosis in patients infected with hepatitis C. This blood test had excellent areas under the receiver operating characteristic curve to predict advanced fibrosis and cirrhosis [128], indicating that endostatin has biomarker potential. One limitation of this study was that patients with advanced fibrosis tended to be older, and renal function measures were not provided [128]. Another study reported serum endostatin levels nearly twice as high in patients with fibrosis as in normal controls. However, this analysis did not describe the disease etiology or the age of the controls in greater detail [80] (Table 3).

Serum endostatin levels were correlated with liver regeneration capacity following hepatectomy in normal mice [113], and with hepatectomy size [113]. Liver regeneration depends on angiogenesis, and both TGF-β and PDGF are essential for liver repair [129]. Endostatin was found to inhibit all of these signaling pathways, while other studies described a role of endostatin in regeneration [80,90,91,98], which calls into question the role of elevated serum endostatin levels in tissue repair.

Currently, it is unclear whether serum endostatin is biologically active. Endostatin purified from human plasma had no effect on endothelial cell proliferation, suggesting that circulating endostatin is inactive with respect to this activity [130].

In summary, the blood and hepatic expression levels of endostatin in chronic liver diseases are greatly understudied compared with other well-known blood proteins.

## 6. Endostatin as a Treatment for HCC

HCC is a vascular tumor in which the pro-angiogenic factor VEGF plays a critical role in disease progression [131]. Along these lines, VEGF-A levels were higher in HCC than in non-tumor tissues [132]. Yet, endostatin/collagen XVIII protein expression, analysed by immunohistochemistry, which did not allow differentiation between these proteins, was reduced [114]. These findings indicate that VEGF activity is greatly increased in HCC. Interestingly, higher endostatin/collagen XVIII expression in tumors was associated with higher VEGF levels and shorter disease-free and overall survival [114], suggesting that low endostatin in tumors is associated with better outcomes. Indeed, poorly differentiated hepatoma cell lines exhibited higher endostatin expression than well-differentiated cell lines. *Collagen XVIII* mRNA expression levels in cell lines correlated with the endostatin protein levels, suggesting that higher *collagen XVIII* is related to increased endostatin protein levels [114].

**Figure 3:**
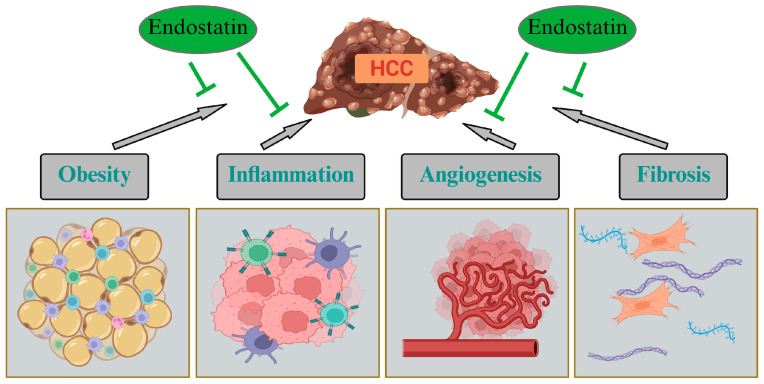
Endostatin and hepatocellular carcinoma (HCC). Endostatin inhibits angiogenesis and reduces inflammation. It may also reverse liver fibrosis and lower body fat, all of which contribute to the development and progression of HCC. Created in BioRender. Pollinger, K. (2026) https://BioRender.com/yjis6qi (accessed on 12 March 2026).

The recombinant endostatin Endostar was approved by China’s State Food and Drug Administration for the treatment of non-small-cell lung cancer in 2005 [90,133]. Recently, the addition of a fragment of the circumsporozoite protein to the C-terminal end of Endostar resulted in more efficient liver targeting. This recombinant protein reduced tumor volume in subcutaneous and orthotopic HepG2 xenograft models much more effectively than Endostar [63]. Tumor microvessel density was reduced by both Endostar and the modified protein, with the latter being more effective. Likewise, the number of apoptotic cells was much higher in mice treated with the modified recombinant protein than in those treated with Endostar [63] (Figure 3).

In an orthotopic HCC model using Hepa1–6 cells, Endostar normalised the angiogenic behaviour of vascular endothelial cells associated with immunity in response to HCC [62] (Figure 3). Hepatic VEGF, hypoxia-inducible factor 1α, CD31, and MMP-2/9 protein levels were reduced [62]. In this study, Endostar also blocked tumor cell infiltration and proliferation [62]. Adenovirus-mediated overexpression of endostatin also inhibited the growth of subcutaneously injected Hep3B cells in nude mice [134]. In contrast, recombinant endostatin had no effect on tumor cell proliferation in vitro but inhibited endothelial cell proliferation [135]. When combined with doxorubicin, a cytostatic drug, endostatin more effectively suppressed the growth of subcutaneous human HepG2 tumors and angiogenesis than either agent alone [135].

Immune cells play a crucial role in HCC development and differ markedly between the livers of HCC patients and healthy controls [136]. The tumor microenvironment plays dual roles in carcinogenesis and disease progression. It facilitates tumor growth, metastasis, and immune evasion while also supporting immunosurveillance [137]. In H22 tumor-bearing mice, hepatic TNF levels were elevated; treatment with Endostar reduced these levels. Endostar enhanced the expression of IL-17, interferon-γ, and CD86, a costimulatory molecule for T-cell activation expressed on monocytes, all of which decreased in the tumors [62]. These results suggest that Endostar therapy normalizes the immune cell landscape in HCC.

Rocha et al. discovered that endostatin and IL-2 synergistically increased the number of CD4 T helper cells and cytotoxic lymphocytes, including natural killer cells and CD8 cells, in renal tumors in mice [138]. In Lewis lung cancer brain metastases in mice, endostatin increased the CD8/CD4 T-cell ratio and polarized M2 macrophages toward the M1 type [139]. M2 macrophages produce VEGF, which is essential for tissue repair [140] and promotes tumor cell growth [141]. However, CD11b+ pro-resolving macrophages, which play a role in resolving inflammation, produce endostatin, thereby lowering angiogenesis [140].

Since VEGF is known to induce an immunosuppressive environment by stimulating the differentiation of regulatory T cells and myeloid-derived suppressor cells while suppressing the differentiation of mature dendritic cells, endostatin may enhance immune cell responses in tumors by blocking VEGF signaling [142].

Several human studies have examined the effectiveness of Endostar as a co-treatment of HCC. Transcatheter arterial chemoembolisation (TACE) is a minimally invasive technique that delivers chemotherapy directly to tumors in the liver. Numerous studies have demonstrated that TACE can significantly improve quality of life and short-term survival in patients with HCC [133]. A recent meta-analysis showed that combining TACE with Endostar was associated with a high overall response and disease control rate. Furthermore, improved survival rates were observed at 6 and 12 months compared to TACE alone. Adverse events associated with the combination of Endostar and TACE were similar to those observed in the TACE-only group [133].

## 7. Current Drugs Used for HCC Treatment

Sorafenib is a multikinase inhibitor that targets various signaling molecules, including VEGF receptors. Approximately 20 years ago, sorafenib was approved and has since been widely used as the primary systemic therapy for patients with advanced, unresectable HCC [143]. However, cancer cells may not respond to sorafenib therapy because they express specific resistance factors. Tumor cells may also become resistant to treatment by acquiring new mutations or undergoing epithelial-to-mesenchymal transition. VEGF induces epithelial-to-mesenchymal transition, which endostatin can block in stages of sorafenib resistance [144]. Thus, endostatin may become a second-line therapy for these patients.

The tumor environment may produce cytokines that activate proliferation pathways [143]. Since resistance to sorafenib is believed to be associated with tumor-associated immune cell activity, combining sorafenib with immunotherapies has been proposed to overcome this resistance. However, immune checkpoint inhibitors targeting the PD-1/PD-L1 pathway have shown limited efficacy in patients with HCC [145]. Combination therapies that incorporate sorafenib have demonstrated significant improvements over sorafenib monotherapy in patients with unresectable or moderately advanced HCC, resulting in prolonged progression-free and overall survival [145].

Atezolizumab, a humanized anti-PD-L1 monoclonal antibody, reactivates exhausted T cells and enhances the body’s immune response against tumors. Atezolizumab combined with bevacizumab—a recombinant human monoclonal antibody that blocks angiogenesis by inhibiting VEGF-A—was recommended as the first-line therapy for unresectable HCC [146]. The activity of Endostar and bevacizumab was comparable in a xenograft zebrafish model of lung cancer [147]. First-line treatments with platinum-based doublet chemotherapy plus either endostatin or bevacizumab had similar anti-tumor activity in patients with metastatic or recurrent lung adenocarcinoma. However, platinum-based doublet chemotherapy + bevacizumab tended to result in worse adverse reactions than platinum-based doublet chemotherapy + endostatin [148]. These studies may encourage further research to evaluate the efficacy of endostatin and atezolizumab as first-line treatment for patients with unresectable HCC.

Endostar was found to inhibit the effect of TGF-β in HSCs [91]. TGF-β acts as a tumor suppressor in the early stages of HCC, but contributes to disease progression in the late stages [149]. Clinical trials are underway to evaluate the impact of TGF-β inhibitors in advanced HCC [150]. TGF-β has multiple functions and affects HSCs, endothelial cells, hepatocytes, and immune cells in HCC tissue [150]. It remains unclear which TGF-β effects are inhibited by endostatin and whether Endostar is effective only at advanced stages of HCC.

The main adverse effects of sorafenib are hypertension, elevated transaminase levels, and proteinuria. These effects did not differ substantially between patients receiving sorafenib monotherapy and those receiving sorafenib plus immunotherapy [145]. The effectiveness of combination therapy with sorafenib and Endostar compared with sorafenib monotherapy has not been tested in patients with HCC. Recombinant endostatin has low toxicity and does not provoke resistance to endostatin [99]. The adverse effects of the endostatin/sorafenib combination may be comparable to those of sorafenib monotherapy.

Endostatin prevents fibronectin binding to integrin α5β1 [33,37], which otherwise promotes tumor growth and angiogenesis in HCC models [151]. Sorafenib, on the other hand, was shown to reduce fibronectin expression in carbon tetrachloride-induced liver fibrosis [152], suggesting that both drugs target fibronectin signaling.

Sorafenib exerts regulatory effects on immune cells, including macrophages, T cells, natural killer cells, and dendritic cells. Sorafenib reduces the number of myeloid-derived suppressor cells and natural killer cells [153,154,155,156]. Sorafenib can increase the differentiation and maturation of dendritic cells by targeting VEGF, but can also reduce interferon α production by these cells [156,157]. Sorafenib alters macrophage polarization and lowers the tumor-promoting activities of M2 macrophages [158]. Endostatin may improve immune cell function in HCC; however, its effects on these cells have been studied far less than those of sorafenib. Preliminary evidence suggests that endostatin is a suitable therapy for patients with HCC, offering the advantage of low toxicity.

## 8. Conclusions

Although endostatin and its therapeutic potential have been known for several decades, studies investigating its expression in different adipose tissues and in the livers of control subjects, obese patients, and patients with various liver diseases are scarce. Endostatin in the bloodstream may serve as a non-invasive biomarker for HCC and liver fibrosis. However, confounding factors, such as spleen size, have yet to be identified. This has not been systematically evaluated in rodents either.

Endostatin is an angiogenesis inhibitor, and several studies support its anti-adipogenic and anti-fibrotic roles. These activities may rely not only on endostatin’s angiostatic function but also on direct effects on adipocytes and hepatic stellate cells. As some analyses show profibrotic effects of endostatin, further studies are needed to clarify the context in which antifibrotic and angiostatic effects occur. Current research suggests that Endostar, a modified form of recombinant endostatin, protects against fibrosis. However, the effects of other forms of recombinant endostatin are unclear. All preclinical studies provided evidence for a protective role of endostatin in HCC. Endostar improved outcomes in patients with HCC when combined with TACE compared with TACE alone. However, anti-angiogenic drugs such as sorafenib or anti-VEGF antibodies like bevacizumab, which both inhibit VEGF signaling pathways, are already used in HCC therapy. Studies comparing Endostar with sorafenib or bevacizumab in patients with HCC are lacking. Endostatin not only inhibits angiogenesis but also reduces inflammation and may reverse liver fibrosis and reduce body fat; all of these activities may prevent HCC development and/or disease progression (Figure 3). Therefore, future studies should **analyse** the effectiveness of endostatin in HCC therapy either alone or in combination with immune checkpoint inhibitors.

## Figures and Tables

**Table 1:** Details of studies that have analyzed blood endostatin levels in controls.

Reference	ELISA	Serum/ Plasma	Endostatin Levels	Results
Sponder et al. [68]	Quantikine ^®^ Human Endostatin Sandwich Enzyme Immunoassay (R&D System Inc., Minneapolis, MN, USA).	Serum	Female control: 167.70 ± 18.19 ng/mL Male control: 118.56 ± 16.41 ng/mL	Higher in females No association with age, body mass index
Zorick et al. [67]	ELISA (Accucyte, Cytimmune Sciences Inc., Rockville, MD, USA)	Serum	Female control: 20.7 + 13.6 ng/mL Male control: 19.3 + 7.3 ng/mL	No sex difference No association with age, body mass index
Kantola et al. [69]	Quantikine Human Endostatin Immunoassay (R&D Systems)	Serum	Female control: 146.5 (113.9–166.4) ng/mL Male control: 121.4 (107.1–166.9) ng/mL Controls < 65 years: 108.1 (101.2–127.0) ng/mL Controls > 65 years: 154.2 (137.7–179.1) ng/mL	No sex difference Higher in controls > 65 years
Silha et al. [65]	Quantikine immunoassays (R&D Systems)	Serum	Female control: ≈120 ng/mL Male control ≈140 ng/mL	No sex difference Higher in obese and overweight females (≈150 ng/mL) than in lean females (≈120 ng/mL)
Larsson et al. [70]	Human endostatin DuoSet ELISA (R&D Systems)	Plasma	Endostatin levels ≈ 40 ng/mL	Positive association with waist circumference, antihypertensive treatment, C-reactive protein, and impaired kidney function (lower ) estimated glomerular filtration rate

**Table 2:** Details of studies that have analyzed the effect of endostatin in liver fibrosis.

Model	Endostatin	Results	Mechanism	Reference
CCl4 + Endostar (20 mg/kg/d for 6 weeks)	Endostar 20 mg/kg/d	**Antifibrotic** ALT, AST ⇓ inflammatory cells ⇓ collagen I, α-SMA, TGF-β1, VEGFR1, VEGFR2 ⇓	Inhibition of activation of HSCs	Chen et al. [90]
HSC-T6 cells cotreatment with VEGF (100 ng/mL) and Endostar for 12 h	Endostar 125, 250, and 500 µg/mL	**Antifibrotic** collagen I ⇓ α-SMA ⇓ TGF-β1 ⇓	Inhibition of VEGF-induced activation of HSCs	Chen et al. [90]
HSC-T6 cells pretreated with endostatin for 1 h and then treated with TGF- β1 (10 ng/mL) or PDGF-BB (200 ng/mL) for 72 h	Endostar 5 µg/mL	**Antifibrotic** collagen I ⇓ F-actin ⇓ α-SMA ⇓ RhoA and ROCK1 ⇓	Inhibition of TGF-β and PDGF-BB induced activation of HSCs	Ren et al. [91]
CCl4-treated mice and recombinant endostatin for 4 weeks	Recombinant endostatin peptides (not described in detail)	**No effect on fibrosis** Sirius Red staining, α-SMA, and CD31 staining did not change	No effect on fibrosis	Zuo et al. [80]
Primary mouse HSCs	Recombinant endostatin 20 and 80 ng/mL	**Profibrotic** α-SMA ⇑ Collagen I ⇑	Profibrotic Activation of α5/β1 integrin	Zuo et al. [80]

(Reduced ⇓; Increased ⇑). The main outcome of these experiments is given in bold.

**Table 3:** Details of studies that have measured serum endostatin levels of patients with chronic liver diseases and patients with hepatocellular carcinoma (HCC). HBV, hepatitis B, HCV, hepatitis C; LC, liver cirrhosis.

Reference	Patients	Sex	Age (Years)	Country	ELISA Serum Levels	Result
Joo et al. [116]	Chronic hepatitis N = 47 Compensated LC N = 26 Decompensated LC N = 11 HCC N = 156 (80% HBV)	173 males 67 females	59.1 ± 11.4	Republic of Korea	Quantikine, R&D Systems Serum endostatin was about 1 ng/mL	Endostatin levels of the patients were similar
Poon et al. [117]	HBV-HCC N = 86 HCV-HCC N = 4 Healthy controls N = 20	76 males 32 females	57.5 (16–75)	China	Cytimmune Sciences Serum endostatin was about 30 ng/mL	Similar serum endostatin levels in controls and patients with HCC
Uematsu et al. [118]	HCC N = 24 (6 HBV, 18 HCV) LC N = 16 (2 HBV, 14 HCV) Healthy controls N = 13	Not defined	56–76 (given for 21 patients with HCC)	Japan	Cytimmune Sciences Serum endostatin was about 30 ng/mL	Similar levels in controls and LC, higher in HCC compared to controls
Sharma et al. [119]	HCC N = 50 LC N = 50 Chronic hepatitis N = 50 Healthy controls N = 50	158 males 42 females	HCC 59 (50–65) LC 47 (40–55) Chronic hepatitis 37 (27–45) Controls 42 (33–48)	India	R&D Systems Serum endostatin was about 100 ng/mL	Similar levels in controls, chronic hepatitis, and LC, and higher in HCC compared to all other groups
Mohsen et al. [120]	HBV or HCV infection ± HCC N = 100 Healthy controls N = 20	Not defined	Not defined	Egypt	Raybiotech, Inc., Peachtree Corners, GA, USA Serum endostatin was about 1 ng/mL in controls and was 90 ng/mL in HCC	Serum endostatin levels in all patients were higher than those of controls. Levels were highest in HCC
Zuo et al. [80]	Liver Fibrosis N = 164 Healthy control N = 30	101 males 63 females	44 (5–79)	China	Cloud-Clone, Katy, TX, USA Serum endostatin was about 200 ng/mL in controls and 400 ng/mL in fibrosis	Endostatin is higher in fibrosis

## Data Availability

No new data were created or analyzed in this study.

## Abbreviations

The following abbreviations are used in this manuscript:

α-SMA	α-smooth muscle actin
CCl4	Carbon tetrachloride
ECM	Extracellular matrix
ERK	Extracellular signal-regulated protein kinase
FAS	Fatty acid synthase
HCC	Hepatocellular carcinoma
HSCs	Hepatic stellate cells
IL	Interleukin
MASH	Metabolic dysfunction-associated steatohepatitis
MASLD	Metabolic dysfunction-associated steatotic liver disease
MMPs	Matrix metalloproteinases
mTOR	Mechanistic target of rapamycin
PDGF	Platelet-derived growth factor
PPAR	Peroxisome proliferator-activated receptor
SREBP	Sterol regulatory element-binding protein
TIMPs	Tissue inhibitors of metalloproteinases
TNF	Tumor necrosis factor
UCP1	Uncoupling protein 1
VEGF	Vascular endothelial growth factor
